# Koch’s New Tuberculin

**Published:** 1897-08

**Authors:** 


**Koch's New Tuberculin.**—Nencki, Maczewski and Logucki (*Presse Medicale*, No. 46), publish some critical remarks on the new preparation of Koch, which merit the attention of the profession. Nencki observed while trying the new tuberculin on a patient, after every injection a considerable reaction with general indisposition, chills, fever, etc. Examining the tuberculin which he used for the injections, he found it containing numerous pneumococci, staphylococci and streptococci. Two other bottles which he opened with every aseptic precaution contained the same bacteria. Inoculated on sterile nutrient media they grew very well. It seemed very probable that during the manufacture of the product, as published by Koch, a contamination could easily occur. In spite of this Koch does not believe that an improvement of this method can be thought of. It is evident that such contaminations can lead to considerable danger for the patient, and it will be necessary that the manufacturers furnish sufficient guarantees for absolute purity of their products by a severe and reliable control; before they can be generally tried as to their efficacy.—*Journal of the American Medical Association.*

---

**An abstract** from a contribution to the *Medical Brief* for October, by Dr. Wm. Murrell, regarding the examination of the outer wrapper in which cigarettes are sold, reads as follows: "I was induced to undertake the investigation from observing symptoms of chronic arsenical poisoning in some of my students who were inveterate cigarette smokers. The tests employed were Reinch and Marsh's, both of which are well known and in constant use. Seventeen different brands were examined, and the labels of six of these contained arsenic, not in minute traces, but in large and readily demonstrable quantities. There is a popular belief that green papers are more likely to contain arsenic than papers of other colors, but this is not the case. One well known American firm which puts up its cigarettes in wrappers of a vivid green color, evidently exercises care in the matter, for not a trace of arsenic was found. On the other hand, a tobacco put up in a bright green wrapper gave abundant indications of the presence of arsenic in the paper. Blue and buff wrappers are as a rule dangerous, and should be avoided."

